# Supplementary figures and images for: Psychometric properties of the general self-efficacy scale among Thais with type 2 diabetes: a multicenter study
Source: PeerJ. 2022 May 23;10:e13398. doi: 10.7717/peerj.13398 (PMC9135036; doi:10.7717/peerj.13398)

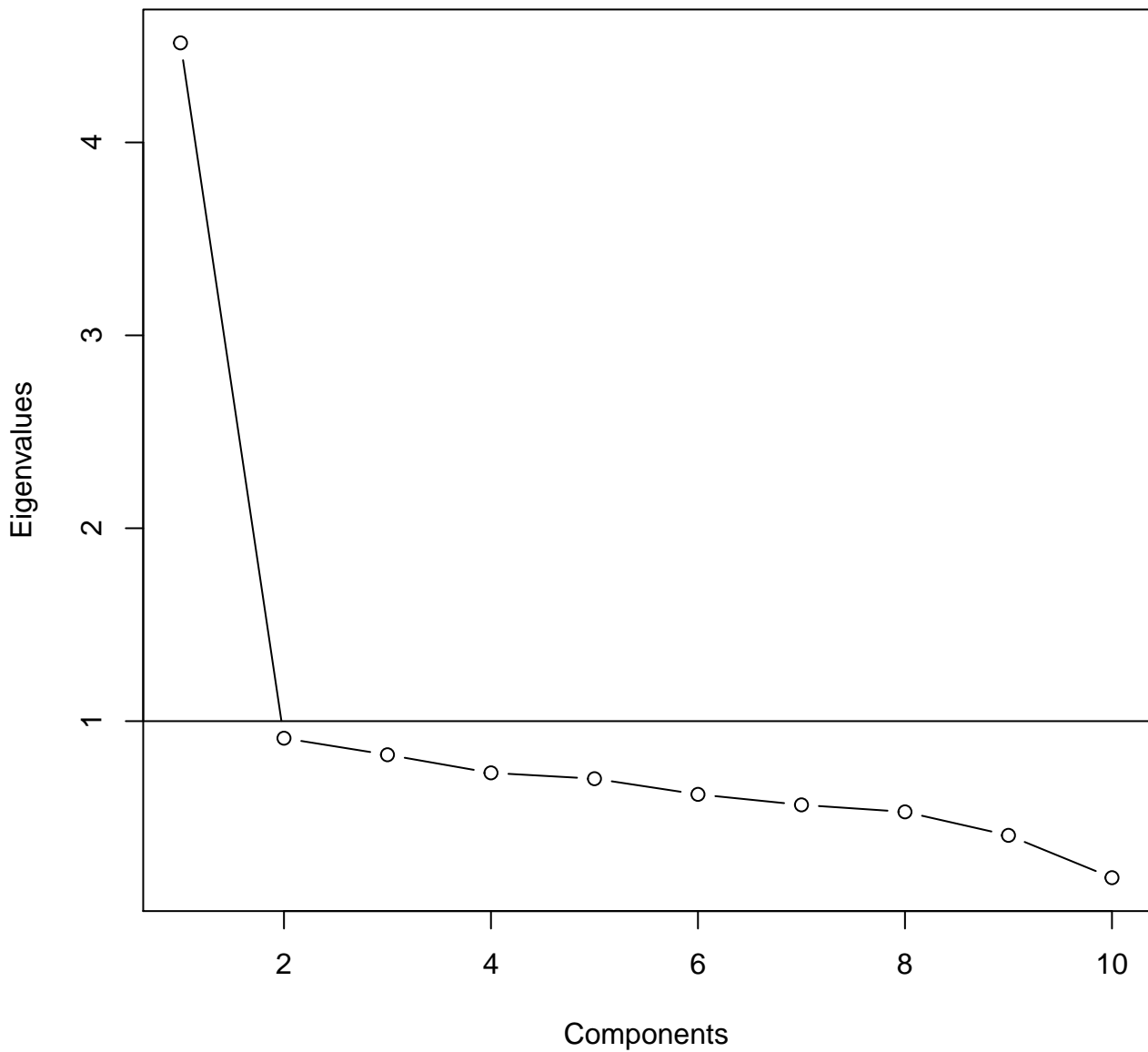

Supplement: Figure S1 — The parallel analysis involves a permutation test to test the hypothesis that each component’s eigenvalue is not greater than 1. [file peerj-10-13398-s001.pdf]
